# Electrical Resistance Prediction for Functionalized Multi-Walled Carbon Nanotubes/Epoxy Resin Composite Gasket under Thermal Creep Conditions

**DOI:** 10.3390/ma12172704

**Published:** 2019-08-23

**Authors:** Wenlong Wang, Xia Yue, He Huang, Chao Wang, Diwei Mo, Yuyan Wu, Qingchun Xu, Chao Zhou, Houyao Zhu, Chunliang Zhang

**Affiliations:** School of Mechanical and Electrical Engineering, Guangzhou University, Guangzhou 510006, China

**Keywords:** smart materials, polymer–matrix composites (PMCs), electrical properties, creep

## Abstract

Carbon nanotube-based conductive polymer composites (CPC) showed great potentials for self-sensing and in situ structural health monitoring systems. Prediction of the long-term performance for such materials would be a meaningful topic for engineering design. In this work, the changing behavior of the long-term resistance of a multi-walled carbon nanotubes/epoxy resin composite gasket was studied under different temperature and loading conditions. Glass transition strongly influenced the resistance behavior of the composite during the thermal creep process. Similar to classical Kelvin–Voigt creep model, a model considering both the destruction and recovery processes of the conductive network inside the CPC was established. The long-term resistance variation can be predicted based on the model, and the results provided here may serve as a useful guide for further design of smart engineering structural health monitoring systems.

## 1. Introduction

In recent years, tremendous progress has been made in the demand for intelligent manufacturing, which brings new opportunities and challenges to the field of signal detection and fault diagnosis. However, applications of many diagnostic techniques are still very complicated, because it is necessary to disassemble and divide the tested parts or adjust the design of the device in order to install additional data-collecting sensors [1,2]. To solve this issue, a smart materials-based in situ sensing and diagnostic method may provide a convenient solution. In mechanical systems, numerous polymer-based parts such as rubber gaskets, hydraulic seals, and aircraft structural composites may simultaneously possess the load-bearing ability and in situ sensing functionality through the modification of carbon nanotubes [3,4].

Since the carbon nanotube was first discovered by Iijima et al. [5], great attention has been drawn due to its excellent mechanical and physical performances. It has been reported that a small volume fraction of carbon nanotubes dispersed in the polymer could form conductive networks, which would enable the composite to have sensing ability to certain thermal and mechanical stimuli [6,7,8]. Based on this characteristic, strain sensor [9] and related damage monitoring systems [10,11,12] have always been a research interest. Fiedler et al. [13] proposed that changes in the conductivity of the composite could be used for damage monitoring. Alexopoulos et al. [14] studied in detail the variation behavior of electrical resistance for carbon nanotube-modified polymer composites in cyclic loading–unloading experiments. Dehghani et al. [15] explored the temperature-resistance characteristics of single-walled and multi-walled carbon nanotube polymer composites. Yin et al. [16] have studied the linear and antisymmetric characteristics of the piezoresistivity for multi-walled carbon nanotubes/epoxy composite. Manufacturing technology such as polydimethylsiloxane (PDMS) molding transfer was reported to fabricate a carbon nanotubes-based polymer composite film [17]. The work by Wang et al. [18] showed that the force sensitivity of the carbon nanotube-based conductive polymer composite could be tuned through alignment of the carbon nanotube and crosslinking degree of polymer chains in the matrix. The capacitive flexible strain sensor fabricated by a single-walled carbon nanotube composite film was reported for human motion detection with high reliability and transparency [19]. Besides, a flexible multi-sensor array was achieved for distributed pressure sensing based on carbon nanotubes/polydimethylsiloxane [20]. 

It can be concluded that because of the unique strain-resistance and temperature-resistance characteristics, the carbon nanotube-modified polymer composite shows fascinating potential in sensing and engineering structure health monitoring. However, limited research has focused on the long-term sensing behavior for carbon nanotube-based conductive composite under both temperature and load-bearing conditions. It has been reported that the carbon nanotube-based polymer composites have a clear resistance creep behavior [21,22], which would directly affect the detection accuracy if no compensation model was applied to the monitoring system. Thus, in the present work, the long-term resistance behavior under different temperature and loading conditions was studied for the carbon nanotube-based polymer composite. Similar to the mechanical creep model of the polymer, a model for the prediction of the variation of its electrical resistance was also established, which may provide a helpful guide for furthering the engineering design of an in-situ sensing or damage monitoring system. 

## 2. Experimental

### 2.1. Composite Preparation

The multi-walled carbon nanotubes (–COOH functionalized MWNTs) between 10–30 µm in length and 10–20 nm in diameter were produced by TIME NANO Co., Ltd (Chengdu, China). The epoxy resin (formulated bisphenol A/F epoxy) and the hardener (formulated liquid aromatic amine) were provided by Tianjin Fusai Science & Technology Co., Ltd (Tianjin, China). 

Considering the conductive property and the material-forming ability of the MWNTs/epoxy composite, the content of MWNTs was selected as 5 wt. %. The composite was prepared as follows: initially, MWNTs were weighted and mixed into acetone with a dispersant (TX-100, Canbo Chemical Co., Ltd, Guangzhou, China) by sonication for 30 min at 600 W. Then, epoxy resin was added and ultrasound dispersed for 30 min. The solution was placed into a heating system (Nobody Material and Technology Co., Ltd, Zhengzhou, China) at 90 °C under vacuum for 2 h in order to remove the acetone solvent. When the mixture cooled to room temperature, hardener with a weight ratio of 100:35 (resin/hardener) and defoamer (X3-6823, Xushi Chemical Technology Co., Ltd, Changzhou, China) with a weight ratio of 0.3:100 (defoamer/resin) were added in order to prevent air bubbles. Followed by mechanically stirring for 5 min, the solution was placed under vacuum for 30 min and then poured into a gasket mold. After solidification at 80 °C for 2 h, the generated gasket sample with a thickness of 2 mm was annealed at 200 °C for 48 h in a vacuum drying oven. 

### 2.2. Scanning Electron Microscope Observation

Field emission scanning electron microscope (FESEM; LEO, Zeiss Co. Ltd., Heidenheim, Germany) was used to observe the cross-section of the MWNTs/epoxy resin composite. The sample was first notched and immersed into nitrogen for over 15 min. Then, it was forced into fracture. Before imaging, the surface for observation was precoated with platinum to enhance conductivity. A high voltage of 5 kV was applied to accelerate the electron beam, and working distance was set around 5 mm with a magnification of 10,000×. Secondary electron emission mode was selected in the observation. The chamber pressure was stabilized around 4 × 10^−4^ Pa after a certain pressure cyclic process. 

### 2.3. Electrical Resistance Measurement during Creep 

**Figure 1:**
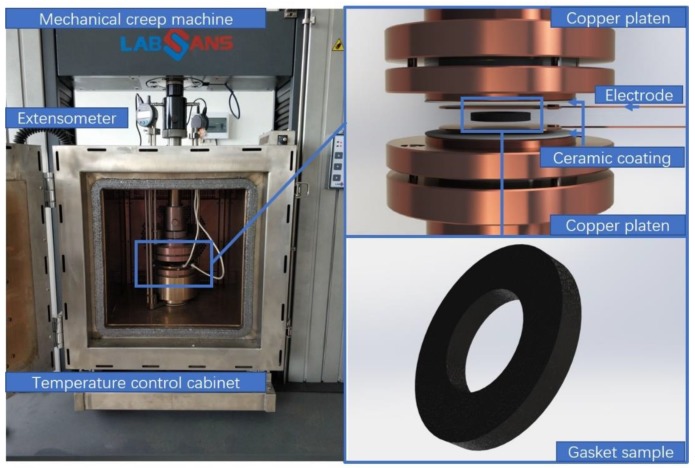
Photo of the mechanical creep test machine equipped with a temperature control cabinet and resistivity measurement. Insulation coating treatment was performed on the contact surfaces of the compressive indenters.

A material testing machine coupled with a temperature-controlled cabinet (Figure 1) was used for measuring the variation of the electrical resistance of the composite during the creep process. The surface of the compressive heads contacting with test sample was coated with an insulative ceramic film to ensure the accuracy of measurement for the samples’ body electrical resistance. Two copper plates contacting the prepared gasket sample were used as electrodes. The temperature of the testing environment was calibrated using a commercial thermocouple (Pt100, Hangzhou SinoMeasure Automation technology Co. Ltd, Hangzhou, China). Four levels of the holding load (1 MPa, 2 MPa, 3 MPa, and 4 MPa) were applied on the gasket sample for 3 h to generate the creep deformation. Under each load, six temperatures at 25 °C, 35 °C, 50 °C, 100 °C, 150 °C, and 200 °C were tested, respectively. The electrical resistance data of the composite was collected by a data acquisition card (PXIe-6366, National Instrument Co., Ltd., Austin, TX, USA) with a sampling rate of 1 kHz. 

### 2.4. Dynamic Thermomechanical Analysis

Dynamic thermomechanical analysis (Q800, TA Instruments Co. Ltd, Newcastle, DE, USA) was used to characterize the glass transition temperature and linear thermal expansivity of the MWNTs/epoxy resin composite. Temperature ranged from 30 to 150 °C with a heating rate of 3 °C/min. Tensile mode was performed at a frequency rate of 1 Hz.

## 3. Results and Discussion

**Figure 2:**
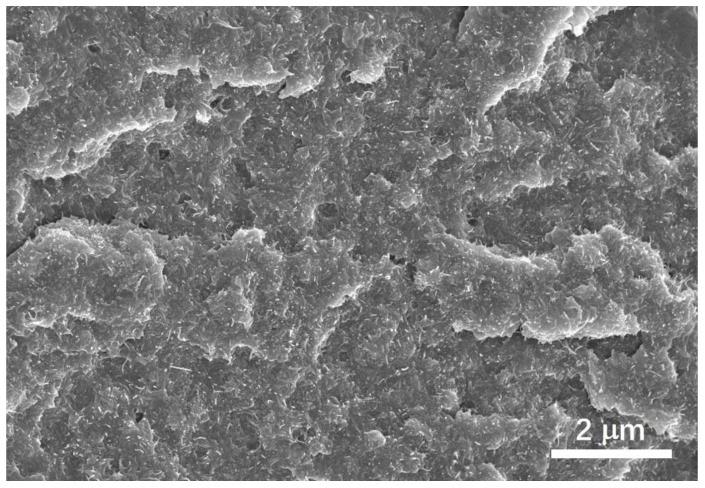
Field emission scanning electron microscope (FESEM) image for the cross-section of the multi-walled nanotubes (MWNT)/epoxy resin composite.

The cross-section of the MWNTs/epoxy resin composite was shown in Figure 2, in which the MWNTs were clearly observed and well dispersed in the epoxy resin matrix at this scale. The preparation process (cryofracturing) for the cross-section of the sample leads to a flat surface without obvious plastic deformation slip and elongation dimple, which indicated the brittle fracture caused by the sample preparation. The voids inside the sample may represent very few unexpunged air bubbles.

**Figure 3:**
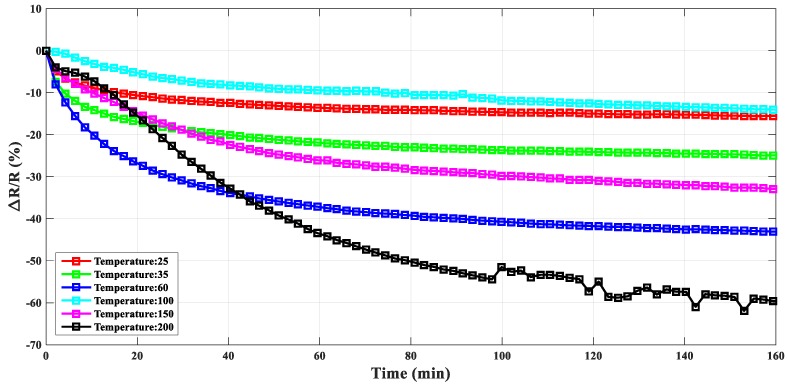
Comparison of the ratio of the electrical resistance variation for the composite gasket under the creep process (with constant holding force: 1000 N) at different temperatures (from 25 °C to 200 °C).

As plotted in Figure 3, during the creep process with stable holding force (1000 N), the ratio of the electrical resistance variation (ΔR/R) of the composite gasket was strongly influenced by temperature. With the increment of the temperature (below 100 °C), the ratio of the electrical resistance variation in the stable stage of the creep process increased to a magnitude of around −0.4. As the temperature increased to 100 °C, the resistance changing ratio (ΔR/R) decreased dramatically, to a value of −0.14, which was slightly larger compared with the response of the resistance variation at room temperature. When the temperature continued to increase, the ratio of the resistance variation increased again. Moreover, the magnitude of the ratio (ΔR/R) reached –0.6. Due to the exponential feature of the creep deformation, the conductive network in the composite gasket was changed, which lead to a similar decreasing trend with time for measuring the electrical resistance-changing ratio at each temperature step. At high temperature (i.e., 200 °C), the fluctuating electrical resistance changing ratio may indicate the damage to the sample caused by the high-temperature creep deformation. Besides, the electrical resistance influenced by temperature for various conductive carbon nanotube–polymer composites [23,24] (namely as positive temperature coefficient (PTC) or negative temperature coefficient (NTC) phenomenon) may be explained by the synergistic effect of the conductive network and electron tunneling effect at different temperatures [25]. During the thermal creep process, the high temperature would increase the slippage of the polymer chain and decrease the electron tunneling potential barrier [26]. In the meantime, the holding load would further enhance the reformation of the conductive network of interconnected agglomerates [27]. Thus, the temperature increment in the creep process could increase the effect of the electrical resistance change.

**Figure 4:**
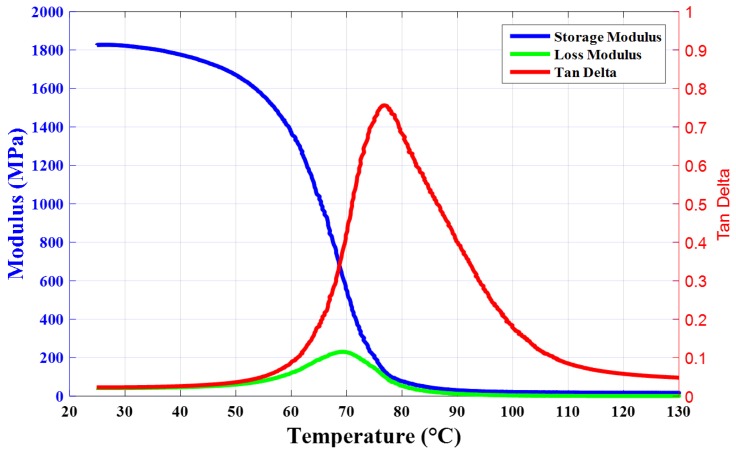
Dynamic thermal mechanical analysis for MWNTs/epoxy resin composite.

Interestingly, as the temperature raised to 100 °C, the resistance changing ratio in the thermal creep process decreased dramatically to a value of approximately 0.15, which was quite similar compared with the value at room temperature. The opposite trend may be caused by a structural change of the conductive network due to thermal creep deformation. As shown in Figure 4, the glass transition behavior of the composite was characterized by dynamical mechanical analysis. The peak of the loss factor ($\tan\left(\delta \right)=\frac{E_{loss}}{E_{storage}}$) indicated that the glass transition temperature of the composite was 77 °C. At the glass transition, the mobility of polymer molecules increased greatly, which would further lead to a large change in the elastic storage modulus [28]. On passing from the glassy to the rubbery state, the storage modulus decreased by three decades from ~1800 MPa to ~5 MPa. The lower viscosity of the polymer matrix at 100 °C may accelerate nanotube secondary agglomeration [29], which resulted in a denser packing of nanotubes inside the agglomerates and correspondingly a low contact resistance and efficient electron transport (i.e., tunneling of electrons) [30]. However, the applied holding compress stress in creep may destroy the conductive filler network. Thus, the different variation trend of the resistance changing ratio (ΔR/R) at 100 °C may be caused by the synergistic effect of higher temperature-induced secondary agglomeration and the destruction of the conductive network caused by creep deformation.

**Figure 5:**
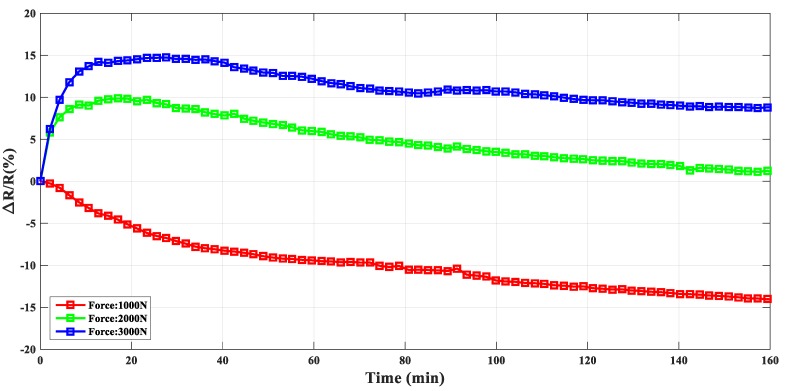
Comparison of the resistance changing ratio with different holding force at 100 °C.

As shown in Figure 5, during the creep process, the electrical resistance changing ratio was influenced by the applied holding compressive force at 100 °C. As the compressive force increased from 1000 N to 3000 N, the resistance transformed though a peak, from increasing (force: 1000 N) with time to decreasing with time (force: 2000 N and 3000 N). The variation of the electrical resistance of the composite was affected by the conductive network formed by carbon nanotube agglomerates, which could be influenced by shear deformation in the creep process [27,31]. The dynamic equilibrium process between the destruction and reformation of the conductive agglomerates may determine the electrical resistance for the composite. Above the glass transition temperature, the enhanced mobility of the polymer chains may intensify the reformation of the agglomerates, which may further lead to the increment of the conductivity [32]. On the contrary, the externally applied force may destroy the carbon nanotube agglomerates and the conductive network. More specifically, a larger external force may lead to the destruction of the agglomerates as the dominating factor, so that the resistivity of the composite would generally increase with time. The syngenetic effect of the destruction and reformation for the conductive agglomerates may play a decisive role in the creep behavior of the resistivity at high temperature. 

**Figure 6:**
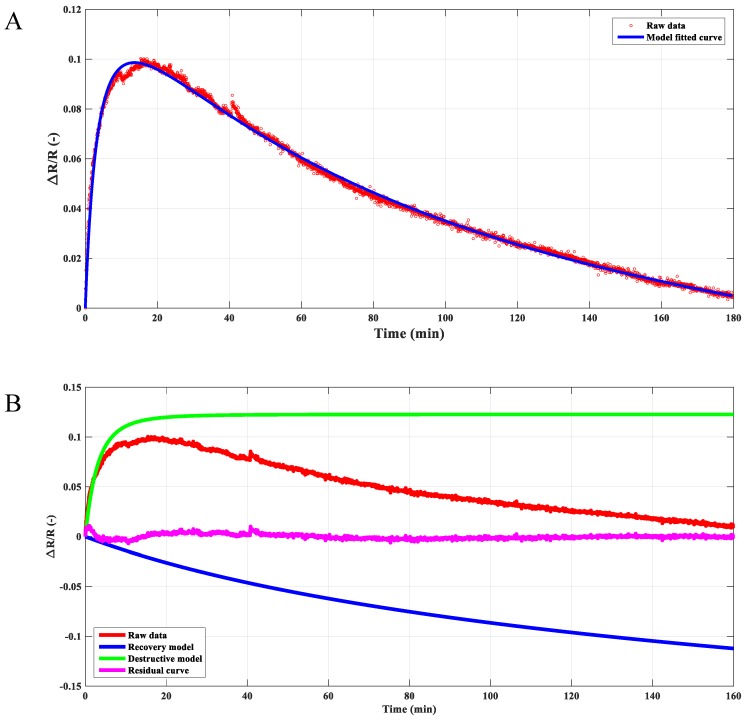
Resistivity variation with time under the compressive force of 2000 N at 100 °C. (**A**) The proposed model matches well with the experimental data. (**B**) The synergistic effect of the destruction and reformation of the conductive network contributed the global resistivity for the composite. Both processes can be extracted based on the proposed model, and little residual error can be found.

For polymer-based composites, creep deformation can be described by the Kevin–Voigt model [33]. Considering the dynamic equilibrium process between the destruction and reformation of the conductive agglomerates, a similar equation was proposed to model the variation behavior of the composite resistivity in the creep process at a given temperature, which can be written as:

(1) $$\frac{\Delta R}{R}=R_{0}\left[1-{\left(1+\frac{t}{\eta_{1}}\right)}^{-\tau_{1}}\right]-R_{1}\left[1-{\left(1+\frac{t}{\eta_{2}}\right)}^{-\tau_{2}}\right]$$

where *t* is the time, and $R_{0}$,$\;R_{1}$, $\eta_{0}$, and $\eta_{1}$ are the fitting parameters. The terms on the right-hand side of Equation (1) represent the destructive and recovery processes of the conductive network in the composite at high temperature, respectively. As an example, the resistivity of the composite under 2000 N at 100 °C can be modeled by Equation (1), as shown in Figure 6A. Furthermore, based on the proposed model, the destruction and reformation process could be extracted, respectively, which were plot in Figure 6B. The recovery model indicated that the resistivity decreased gradually with time because of the reformation of the secondary conductive agglomerates at high temperature. In contrast, the degree of the destruction for the conductive network increased along with the applied pressure and eventually reached stability. The competition mechanism between the destruction and the reformation of the conductive paths inside the composite contributed to the global behavior of the composite resistivity. Similar results can also be found in the work of Ingo et al. [34]. What’s more, the residual error can be obtained through the raw experimental data subtracted by the predicted model. The peaks appeared in the residual error curve may indicate that structural defaults were generated in the composite during the creep process.

**Figure 7:**
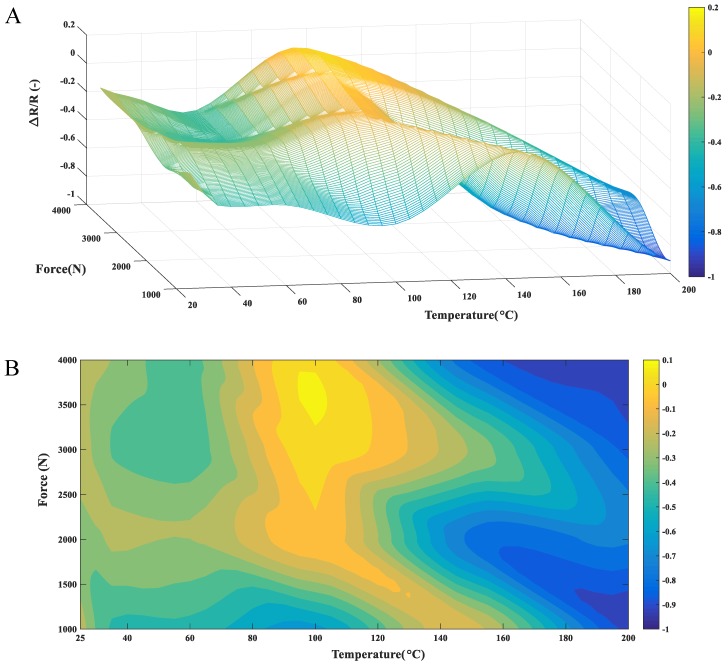
Contour plot for long-term predictions of the resistance variation ratio under different loading and temperature conditions in two perspectives: (**A**) oblique view. (**B**) Top view.

Long-term stabilities of the performances of the polymer-based composite form the major limitation for engineering applications. Based on the proposed model, resistance in long-time service condition could be predicted. All the parameters in the model were obtained by the MATLAB curve fitting toolbox with the experimental data, which were carried out under specific force and temperature conditions during the short time (3 h) resistance creep test process. Here, a life span of 10 years was selected for the gaskets made of multi-walled carbon nanotubes/epoxy composite. The predicted variation ratio of the resistivity was plotted in Figure 7. The changing ratio of the resistance was directly related to the loading conditions and the external temperatures in the service life. Depending on the predicted values, the long-term behavior of the resistance for the composite may be divided into three zones, as marked in Figure 7B. When the temperature was less than 80 °C, the long-term resistance changing ratio was within the range of −0.2 to −0.5, which may be used as the safe area for engineering design. The resistance was generally decreased due to the secondary reformation of the conductive agglomerates. The second area in the middle of the chart may be named the structural transition zone. When the external temperature neared the glass transition temperature of the composite, the long-term resistance increased compared with the initial state. This increment of the long-term resistance may be caused by the strong structural variation of the conductive path, which would be more intense under the simultaneous action of load and high temperature. The third area on the right part of the chart was denoted as the high-temperature softening zone. The long-term predicted value reached nearly −0.9, which indicated that the composite gasket was almost in failure mode after a 10-year life span service. The value was reasonable when the composite was submitted in extremely serious loading conditions for a long period. The chart provided here would be helpful for engineering design with the MCNTs/epoxy resin conductive polymer composite, particularly in the applications, such as the stress/strain sensors and other in situ structural health monitoring systems. 

## 4. Conclusions

The electrical resistance of the studied multi-walled carbon nanotubes/epoxy resin composite was changing all the time during the thermal creep process, which may be explained by the dynamic equilibrium between the destruction and recovery processes of the conductive network inside the composite. A model was established and could be used to well describe the resistance variations during the thermal creep process. Based on the proposed model, the long-term resistance changing ratio under different external temperatures and loading conditions (different amounts of applied compressive forces) can be predicted, and it may serve as a helpful design guide for further sensing and self-detection applications with multi-walled carbon nanotubes/epoxy resin composite.
